# Clinical features and mutational analysis of X-linked agammaglobulinemia patients in Malaysia

**DOI:** 10.3389/fimmu.2023.1252765

**Published:** 2023-09-22

**Authors:** Chai Teng Chear, Intan Hakimah Ismail, Kwai Cheng Chan, Lokman Mohd Noh, Asiah Kassim, Amir Hamzah Abdul Latiff, Sandeep Singh Gill, Nazatul Haslina Ramly, Kah Kee Tan, Charlotte Sundaraj, Chong Ming Choo, Sharifah Adlena Syed Mohamed, Mohd Farid Baharin, Amelia Suhana Zamri, Sharifah Nurul Husna Syed Yahya, Saharuddin Bin Mohamad, Adiratna Mat Ripen

**Affiliations:** ^1^ Primary Immunodeficiency Unit, Allergy and Immunology Research Centre, Institute for Medical Research, National Institutes of Health, Ministry of Health, Shah Alam, Selangor, Malaysia; ^2^ Clinical Immunology Unit, Department of Paediatrics, Faculty of Medicine and Health Sciences, Universiti Putra Malaysia, Serdang, Malaysia; ^3^ Pediatric Department, Penang General Hospital, Ministry of Health, George Town, Penang, Malaysia; ^4^ Pediatric Department, Tunku Azizah Hospital (Women and Children Hospital Kuala Lumpur), Ministry of Health, Kuala Lumpur, Malaysia; ^5^ Allergy and Immunology Centre, Pantai Hospital, Kuala Lumpur, Malaysia; ^6^ Pediatric Department, Hospital Wanita Dan Kanak-Kanak Sabah, Ministry of Health, Kota Kinabalu, Sabah, Malaysia; ^7^ Pediatric Department, Perdana University and Royal College of Surgeons in Ireland (PURCSI), School of Medicine, Perdana University, Kuala Lumpur, Malaysia; ^8^ Pediatric Department, Hospital Putrajaya, Ministry of Health, Putrajaya, Malaysia; ^9^ Pediatric Department, Hospital Sultan Abdul Halim, Ministry of Health, Sungai Petani, Kedah, Malaysia; ^10^ Pediatric Department, Hospital Sultanah Aminah, Ministry of Health, Johor Bahru, Johor, Malaysia; ^11^ Institute of Biological Sciences, Faculty of Science, Universiti Malaya, Kuala Lumpur, Malaysia; ^12^ Centre of Research in Systems Biology, Structural Bioinformatics and Human Digital Imaging (CRYSTAL), Universiti Malaya, Kuala Lumpur, Malaysia

**Keywords:** X-linked agammaglobulinemia, XLA, Bruton’s tyrosine kinase, BTK protein, BTK mutation, *de novo* mutation, COVID-19

## Abstract

**Background:**

Bruton’s tyrosine kinase (BTK) is a cytoplasmic protein involved in the B cell development. X-linked agammaglobulinemia (XLA) is caused by mutation in the *BTK* gene, which results in very low or absent B cells. Affected males have markedly reduced immunoglobulin levels, which render them susceptible to recurrent and severe bacterial infections. Methods: Patients suspected with X-linked agammaglobulinemia were enrolled during the period of 2010-2018. Clinical summary, and immunological profiles of these patients were recorded. Peripheral blood samples were collected for monocyte BTK protein expression detection and *BTK* genetic analysis. The medical records between January 2020 and June 2023 were reviewed to investigate COVID-19 in XLA.

**Results:**

Twenty-two patients (from 16 unrelated families) were molecularly diagnosed as XLA. Genetic testing revealed fifteen distinct mutations, including four splicing mutations, four missense mutations, three nonsense mutations, three short deletions, and one large indel mutation. These mutations scattered throughout the *BTK* gene and mostly affected the kinase domain. All mutations including five novel mutations were predicted to be pathogenic or deleterious by *in silico* prediction tools. Genetic testing confirmed that eleven mothers and seven sisters were carriers for the disease, while three mutations were *de novo*. Flow cytometric analysis showed that thirteen patients had minimal BTK expression (0-15%) while eight patients had reduced BTK expression (16-64%). One patient was not tested for monocyte BTK expression due to insufficient sample. Pneumonia (n=13) was the most common manifestation, while *Pseudomonas aeruginosa* was the most frequently isolated pathogen from the patients (n=4). Mild or asymptomatic COVID-19 was reported in four patients.

**Conclusion:**

This report provides the first overview of demographic, clinical, immunological and genetic data of XLA in Malaysia. The combination of flow cytometric assessment and *BTK* genetic analysis provides a definitive diagnosis for XLA patients, especially with atypical clinical presentation. In addition, it may also allow carrier detection and assist in genetic counselling and prenatal diagnosis.

## Introduction

X-linked agammaglobulinemia (XLA; OMIM# 300300 and 300755) is a congenital immunodeficiency characterized by an early arrest in B cell differentiation, resulting in a marked reduction of peripheral B cells with a profound deficiency in all immunoglobulin isotypes (1). The prevalence of XLA was reported to be 1 in 300,000 people in a Japanese series (2). Patients with XLA have increased susceptibility to bacterial infections, predominately affecting respiratory, skin and gastrointestinal system (3, 4). To prevent infections, XLA patients are treated with either intravenous or subcutaneous immunoglobulin replacement therapy (5, 6).

XLA is caused by mutation in the Bruton’s tyrosine kinase (*BTK*) (7, 8). The *BTK* gene is located on the chromosome X, Xq21.3-Xq22, encompassing 37.5 kb genomic DNA (9). *BTK* gene contains 19 exons that encodes for a cytoplasmic protein tyrosine kinase, which is important in the B cell differentiation. BTK protein contains several domains: an N-terminal pleckstrin homology (PH) domain, Tec homology (TH) domain, Src homology 3 (SH3) domain, Src homology 2 (SH2) domain, and a C-terminal kinase (TK or SH1) domain (10). The PH domain is important for membrane localization, while TH domain is involved in the protein folding and activity regulation. The SH3 and SH2 domains are important for protein-protein interactions, and SH1 domain is the catalytic domain for tyrosine phosphorylation (11).

According to a BTK database, *BTK* gene mutations scattered throughout the gene, affecting both coding and non-coding regions (12). Patients with missense mutation in a non-conserved residue or mutation involving non-coding regions have milder phenotypes. On the other hand, patients with non-sense mutation involving premature stop codon have severe phenotypes (13). Some patients with premature stop codon might have early onset of disease, but some might have a later onset of disease (14). Therefore, there is no clear correlation between genotype and phenotype observed in XLA patients. Thus far, there have been many reports on XLA from East Asia regions (3, 15–23), while a few XLA reports were from Southeast Asia (15, 24). In addition, information on XLA incidence in Malaysia is scarce (25–30). Hence, the objective of this study was to describe the genotypes and clinical phenotypes of Malaysian patients with XLA.

## Materials and methods

### Patients and study design

Our laboratory serves as a national referral center for primary immunodeficiency diseases including X-linked agammaglobulinemia (XLA). In this study, 22 male patients (age ranged 14 months old to 35.4 years old) with XLA-compatible phenotypes from 16 families were recruited from 2010 to 2018. XLA was clinically diagnosed according to the European Society for Immunodeficiencies criteria (31): having less than 2% peripheral B lymphocytes, low to undetectable levels of serum immunoglobulins, and increased susceptibility to bacterial infections. The clinical (age of onset, age of diagnosis and clinical presentations) and immunological data (B cells and immunoglobulin levels) of the patients included in this paper were retrieved from medical records. The mother, brother(s) and sister(s) of the same families were screened, when available. Written informed consent was obtained from parents or patients for genetic testing. This study was approved by the Medical Research and Ethics Committee, Ministry of Health Malaysia (KKM/NIHSEC/08/0804/P12-364).

### Monocyte BTK expression by flow cytometry

Monocyte BTK expression was assessed in most of the patients using flow cytometer, as previously described (26). Monocyte BTK expression was examined on either BD FACSCalibur™ (before year 2013) or BD FACSCanto™ II (since year 2013). Patient P10 was critically ill during recruitment, the obtained blood sample was insufficient to examine the monocyte BTK expression and B cell level. Fifty control samples obtained from unrelated healthy adult volunteers were also subjected to evaluation of monocyte BTK expression.

### Genetic testing

Total RNA was extracted from whole blood with QIAamp^®^ RNA blood mini kit (Qiagen Inc, Hilden, Germany) according to the manufacturer’s instructions. Total RNA (1 µg) was primed with random primers (Promega, Madison, USA) and then reverse transcribed into cDNA using Superscript™ II first strand reverse transcriptase (Invitrogen, Lithuania). The genomic DNA was extracted from the peripheral blood mononuclear cells (PBMCs) using QIAamp^®^ DNA mini kit (Qiagen Inc, Hilden, Germany) following manufacturer’s instructions.

All patients (n=22) and three asymptomatic brothers were subjected to *BTK* genetic testing. Genetic carrier screening was also performed among 14 mothers and 12 sisters. The *BTK* gene (NM_000061.2) was amplified from the subject’s cDNA (1 µl) using seven overlapping primers (32) and 1.25 U AmpliTaq DNA polymerase (Applied biosystems, CA, USA) with 56°C annealing temperature. Each PCR product was separated by 2% agarose gel electrophoresis. For the PCR product that shown single band on agarose gel, PCR clean-up was performed before subjecting to bi-directional Sanger sequencing. As for the PCR product that shown multiple bands on agarose gel, each band was purified from the agarose gel before subjecting to bi-directional Sanger sequencing. On the other hand, each mutation identified in the cDNA level was further verified by amplifying the intronic regions (NG_009616.1) flanking the mutation site using genomic DNA (200 ng) and specific primers (33). The PCR products were also sequenced in both directions. To verify the mutation with a large indel mutation in the patients P3 and P4, a long-range PCR was performed on their genomic DNA using specific primers (33). Molecular cloning and primer walking were subsequently performed.

### *In silico* prediction of variant pathogenicity

The nomenclature of all sequence variants was assessed using Mutalyzer (34) as recommended by the Human Genome Variation Society (HGVS). All variants were then subjected to *in silico* predictions to evaluate the variant pathogenicity. The pathogenicity of the missense mutations were analyzed using prediction tools such as SIFT (https://sift.bii.a-star.edu.sg) and PolyPhen-2 (http://genetics.bwh.harvard.edu/pph2/). All variants except for those affecting an exon/intron boundary were also subjected to MutationTaster2 (https://www.mutationtaster.org/) for pathogenicity prediction. Provean (http://provean.jcvi.org) was used to predict the impact of an amino acid substitution or indel on the biological function of a protein. The pathogenicity of all variants was further evaluated using VarSome server (https://varsome.com).

### COVID-19 infection in XLA

The occurrence of COVID-19 in XLA was investigated. The medical records of 14 XLA patients were reviewed between January 2020 and June 2023.

## Results

### Clinical and immunological characteristics of XLA patients

**Figure 1:**
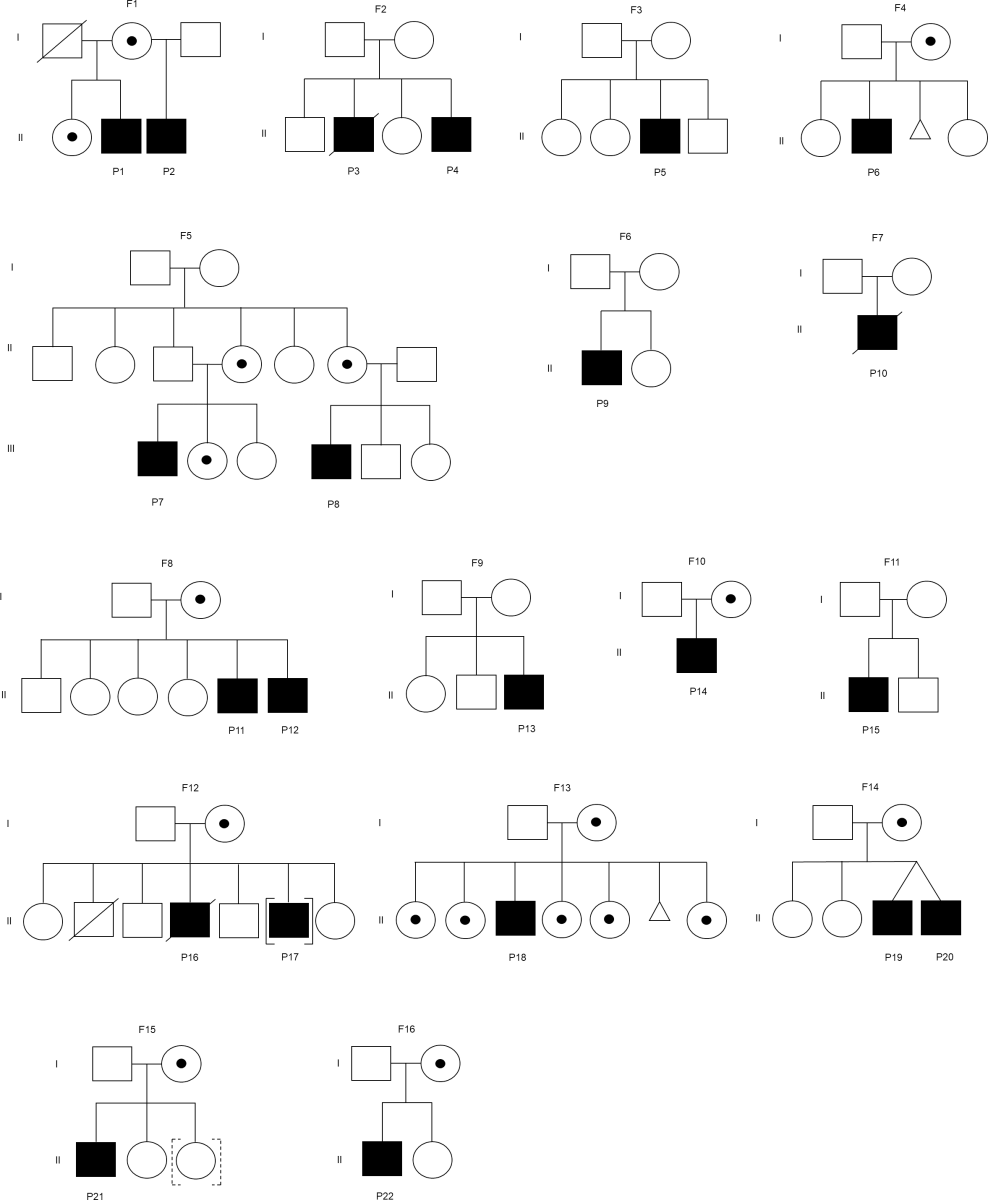
Family pedigree of the patient. F represents a family. Square indicates male, circle indicates female, circle with dot indicates carrier, slash indicates deceased, triangle indicates miscarriage, [] indicates adopted out, and dashed [] indicates adopted in. The mothers in the families (F2, F6, and F7) were unavailable for carrier screening. The mothers in the families (F3, F9, and F11) were not disease carriers.

Twenty-two XLA patients from 16 families were recruited in this study (**Figure 1**). The median age of onset of symptoms was 12 months (range 0.3-154 months) (**Table 1**). The median age of diagnosis was 48 months (range 6-188 months). All patients except P10 had markedly reduced (0-1%) CD19+ B cells. Most of these patients (91%) exhibited very low serum IgG level. In addition, 95% and 64% patients had low IgA and IgM level as compared to the same age group, respectively. Comparing the clinical presentations, pneumonia was the most common manifestation among the XLA patients (n=13), followed by otitis media (n=12), upper respiratory tract infection (n=5), cellulitis (n=4) and gastroenteritis (n=4).

**Table 1:** The laboratory investigations and clinical characteristics of XLA patients.

Patient ID	Present age	Age of onset (year)	Age at diagnosis (year)	IgG (g/l)	IgA (g/l)	IgM (g/l)	B cells (%)	Monocyte BTK expression (%)	Clinical presentations
P1	6Y10M	1Y	6Y9M	0.41	0.48	<0.12	0	6	Recurrent bronchopneumonia, recurrent otitis media, asthma, bronchiectasis
P2	1Y5M	10M	1Y5M^€^	0.61	0.10	0.12	0	18	Recurrent lymphadenitis, pneumonia. Positive family history of XLA
P3	12Y10M	7M	10Y	0.31	TLTD	0.44^ƭ^	0	1	Recurrent pneumonia, bronchiectasis, died of severe pneumonia
P4	6Y9M	2M	3Y10M	0.13	TLTD	0.04	0	1	Recurrent URTI, frequent cough and fever. Positive family history of XLA
P5	2Y	1Y	1Y10M^€^	6.20^ƭ^	<0.38	<0.26	0	14	Pneumonia, Otitis media, asthma, necrotizing fasciitis, failure to thrive, bronchiectasis
P6	5Y	8M	4Y	<2.11	<0.38	<0.26	0	0	Pneumonia, chronic suppurative otitis media, otitis externa, left gluteal cellulitis, thigh and perianal abscesses, chronic subacute arthritis, knee synovitis
P7	12Y8M	2Y	6Y9M	0.91^*^	TLTD^*^	0.11^*^	0	0	Otitis media, recurrent sinusitis, meningoencephalitis
P8	2Y5M	2M	1Y9M	<0.17^*^	<0.02^*^	0.21^*,ƭ^	0	11	Pneumonia, URTI, otitis media, bronchiectasis. Positive family history of XLA
P9	7Y10M	1Y	4Y	0.30	0.05	0.05	1	8	Pneumonia, URTI, gastroenteritis, bronchiectasis
P10	35Y5M	NA	10Y	3.99	TLTD	TLTD	NA	NE	URTI, cellulitis, cholecystitis, diarrhea, died of sepsis
P11	5Y3M	1Y	4Y10M	0.42	0.05	0.81^ƭ^	0	4	Otitis media, oropharyngeal abscess, preseptal cellulitis, frequent abscesses and cellulitis
P12	6Y2M	5Y	6Y2M^€^	1.32	<0.02	0.20	0	5	Otitis media, meningitis, AGE, acute rhinosinusitis, bilateral conjunctivitis, alpha thalassemia trait. Positive family history of XLA
P13	12Y8M	10Y	11Y7M	<0.19^*^	0.07^*^	0.43^*,ƭ^	0	57	Recurrent pneumonia, pulmonary TB, bronchiectasis with right lung fibrosis secondary to pulmonary tuberculosis, septic arthritis, multiple dental carries, thoracic scoliosis. History of right hip TB with history of relapse
P14	1Y2M	6M	6M	1.28	0.66^ƭ^	<0.23	0	15	Perianal ulcer, left thigh abscess, AGE with severe dehydration
P15	11Y	4Y	10Y9M^€^	<0.17	<0.02	<0.21	0	1	Pneumonia, otitis media, sinus venosus atrial septal defect, septic arthritis, bronchiectasis
P16	6Y	3Y1M	4Y	<0.17^*^	<0.02^*^	<0.1^*^	0	30	Pneumonia, empyema thoracis, eczema, failure to thrive, bronchiectasis, died due to complication of cerebral palsy. An elder brother died due to sepsis at age of 3 years old
P17	3Y4M	2Y	2Y10M	2.88	<0.02	0.16	0	62	Recurrent sinopulmonary infection, otitis media, gastroenteritis, meningitis. Positive family history of XLA
P18	16Y1M	12Y10M	15Y8M^€^	5.48	<0.10	1.58^ƭ^	0	20	Recurrent pneumonia, peritonitis secondary to interloop bowel abscess with adhesion, hand foot mouth disease, bronchiectasis
P19	2Y6M	D9	2Y3M	0.59^*^	<0.02^*^	0.51^*,ƭ^	0	58	Pneumonia, otitis media, hand foot mouth disease, skin abscess
P20	2Y6M	D15	2Y3M	0.43^*^	<0.02^*^	0.31^*,ƭ^	0	64	Pneumonia, otitis media, urinary tract infection, skin and scalp abscess, bronchiectasis
P21	7Y11M	2Y	5Y5M	8.80^ƭ^	<0.02	0.38^ƭ^	0	18	Left otitis media, ecthyma gangrenosum, septic shock with left endophthalmitis, staphylococcus toxic shock syndrome with varicella infection
P22	5Y8M	1Y	3Y1M	0.39^*^	<0.02^*^	<0.1^*^	0	1	Recurrent URTI, cellulitis, perianal abscess, thigh abscess, necrotizing fasciitis

Y, year; M, month; D, day; *, Ig levels before receiving the first IVIG therapy; TLTD, too low to be detected; NA, not available; ^ƭ^, isotype is normal for age; NE, not examined; Present age, the age when the genetic test is done; Age at diagnosis, the age when the first dose of IVIg received; ^€^, when the monocyte BTK expression was assessed; XLA, X-linked agammaglobulinemia; URTI, upper respiratory tract infection; AGE, acute gastroenteritis; TB, tuberculosis.

### Monocyte BTK protein expression assessment by flow cytometry

**Figure 2:**
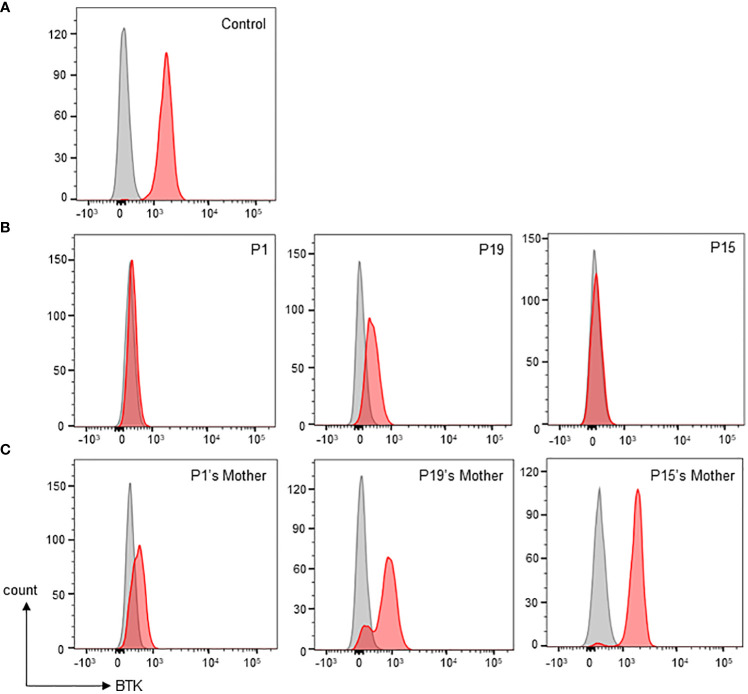
Monocyte BTK expression evaluation by flow cytometric assay. **(A)** Normal control showed presence of BTK in monocytes. Grey shaded histogram represents isotype control; red shaded histogram represents BTK expression. **(B)** Representative patients with various types of monocyte BTK expression. Patient P1 (left panel) with almost complete BTK deficiency in monocytes. Patient P19 (middle panel) with partial BTK deficiency in monocytes. Patient P15 (right panel) with almost complete BTK deficiency in monocytes. **(C)** Representative mothers with various patterns of monocyte BTK expressions. The P1’s mother (left panel) with mosaic pattern of BTK expression in monocytes. The P19’s mother (middle panel) with a different mosaic pattern of BTK expression in monocytes. The P15’s mother (right panel), was not a carrier, had a normal monocyte BTK expression.

Monocyte BTK protein expression was assessed in all patients except P10 (**Table 1**). Thirteen patients had complete/almost complete BTK deficiency, while eight patients had partial BTK deficiency (**Figure 2**). On the other hand, normal controls showed intense BTK expression (76-100% positive) in the monocytes. BTK expression on monocytes were also examined in some of the patient’s family members (**Supplementary Table 1**). Thirty-two family members including 15 mothers, four brothers, and 13 sisters were recruited for monocyte BTK expression assessment. Eleven mothers and seven sisters showed mosaic expression, while normal BTK expression was observed in four mothers, six sisters, and four brothers.

### *BTK* genetic analysis

The *BTK* genetic analysis encompassing exons 1-19 in the cDNA level was carried out to confirm the diagnosis. Out of the 15 unique mutations, one mutation was located in the PH domain (n=1), nil mutation in the TH domain, one mutation in the SH3 domain (n=3), two mutations in the SH2 domain (n=4), one mutation involving both SH3-SH2 domains (n=2) and 10 mutations in the kinase domain (n=12) (**Supplementary Table 3**). Among these mutations, five were novel, including a large indel mutation (NC_000023.11:g.101358809_101361213delins[(302); NG_052969.1:g.788322_791554;GT]), two substitutions (c.1181C>A, and c.1559G>T), a short indel mutation (g.33640-33661delinsTG) and a splicing mutation (c.1103-2A>G) (**Table 2**). Interestingly, the *BTK* genetic analysis in genomic DNA level revealed that seven of these 15 unique mutations were attributed to substitution or deletion of its respective splice site(s). Genetic screening was also performed to verify the carrier or disease status of the family members (n=29). Eleven mothers (11/14) and seven sisters (7/12) were carriers to the disease. In addition, three *de novo* mutations were identified (c.1181C>A, c.1559G>T, and c.215del). The mothers in the three families (F2, F6, and F7) were unavailable for carrier screening. Genetic screening was performed on an asymptomatic brother in the family F8, and two asymptomatic brothers in the family F12. Genetic analysis demonstrated that none of these asymptomatic brothers (0/3) inherited the mutation.

**Table 2:** Novel *BTK* mutations identified in XLA patients.

Family no.	Patient ID	Localization	Domain affected	Nucleotide change in genomic DNA	Nucleotide change in cDNA level	Predicted codon change	Type of mutation
F2	P3	Intron 7-10	SH3,SH2	NC_000023.11:g.101358809_101361213delins[(302);NG_052969.1:g.788322_791554;GT]	c.589_894del	p.Ile197_Glu298del	Indel
F2	P4	Intron 7-10	SH3,SH2	NC_000023.11:g.101358809_101361213delins[(302);NG_052969.1:g.788322_791554;GT]	c.589_894del	p.Ile197_Glu298del	Indel
F3	P5	Exon 14	Kinase	g.34273C>A	c.1181C>A	p.Ser394*	Nonsense mutation
F9	P13	Exon 15	Kinase	g.35166G>T	c.1559G>T	p.Arg520Leu	Missense mutation
F12	P16	Intron 12 & Exon 13	SH2	g.33640-33661delinsTG	c.1103_1129del	p.Gly368_Pro376del	Splicing
F12	P17	Intron 12 & Exon 13	SH2	g.33640-33661delinsTG	c.1103_1129del	p.Gly368_Pro376del	Splicing
F14	P19	Intron 12	SH2	c.1103-2A>G	c.1103_1115del; c.1102_1103ins[NG_009616.1: g.33472_33639;GG]	p.Leu369Serfs*30; p.Gly368Valfs*32	Splicing
F14	P20	Intron 12	SH2	c.1103-2A>G	c.1103_1115del; c.1102_1103ins[NG_009616.1: g.33472_33639;GG]	p.Leu369Serfs*30; p.Gly368Valfs*32	Splicing

### *In silico* prediction of variant pathogenicity

All novel variants were predicted as pathogenic, deleterious or disease causing by at least two *in silico* prediction tools (**Table 3**). All missense mutations (n=4) were predicted to have pathogenic effects by SIFT, Polyphen-2, MutationTaster2, Provean and Varsome prediction tools (**Supplementary Table 4**). MutationTaster2 was able to predict the impact of variants (n=10) except those indel or splicing mutations. Provean was able to predict the impact of the variants with missense and indel mutations (n=9). Varsome was able to predict the impact of all variants (n=15) regardless the type of mutation. All variants were predicted as pathogenic (n=13) or likely pathogenic (n=2) by Varsome prediction tool.

**Table 3:** *In silico* functional impact predictions of the identified novel *BTK* mutations.

Patient ID	Mutation	SIFT	Polyphen-2	MutationTaster2	Provean	Varsome
P3	c.589_894del^£^; I197_E298del^¥^	–	–	–	Deleterious	Pathogenic
P4	c.589_894del^£^; I197_E298del^¥^	–	–	–	Deleterious	Pathogenic
P5	c.1181C>A^ɤ,£^	–	–	Disease causing	–	Pathogenic
P13	c.1559G>T^ɤ,£^; R520L^§,ρ,¥^	Affect protein function	Probably damaging	Disease causing	Deleterious	Pathogenic
P16	c.1103_1129del^ɤ,£^; G368_P376del^¥^	–	–	Disease causing	Deleterious	Pathogenic
P17	c.1103_1129del^ɤ,£^; G368_P376del^¥^	–	–	Disease causing	Deleterious	Pathogenic
P19	c.1103_1115del^ɤ,£^	–	–	Disease causing	–	Pathogenic
P20	c.1103_1115del^ɤ,£^	–	–	Disease causing	–	Pathogenic

The mutation nomenclature used as the input for SIFT^§^, Polyphen-2^ρ^, MutationTaster2^ɤ^, Provean^¥^ and Varsome^£^ prediction tool. –, not applicable.

### COVID-19 infection in XLA

COVID-19 infection was documented in four of the XLA patients (**Supplementary Table 2**). A patient (P15) had an episode of COVID-19 category 1, while three patients (P5, P17 and P20) had an episode of COVID-19 category 2.

## Discussion

In the present study, the clinical, immunological, and genetic features of 22 Malaysian patients with XLA were described. Approximately 60% of the patients manifested symptoms within the first year of life. The median age at onset of symptoms was 12 months. While, the median age at diagnosis was 48 months with a median delay in diagnosis of 26.7 months. The median age at diagnosis in our cohort was similar with the study from Iran (35), however, higher than other studies in North Africa (36 months) and Argentina (42 months) (14, 36), and lower than the studies in Taiwan (60 months) and China (84 months) (21, 23). In some countries, kappa-deleting recombination excision circle (KREC) screening in newborns is implemented to detect XLA at birth (37–40). The use of KREC assay in newborn screening may reduce diagnostic delay.

Our analysis revealed that XLA patients exhibited various clinical presentations, ranging from common to atypical presentations. Most XLA patients in this study exhibited common presentations such as respiratory infections, arthritis, skin infections, meningitis, and encephalitis. A minority of XLA patients exhibited atypical clinical presentations such as necrotizing fasciitis caused by rare fungus (n=1) (27), and tuberculosis caused by *Mycobacterium tuberculosis* (n=1). Apart from the manifestation of tuberculosis, some patients with *BTK* mutation affecting the kinase or SH2 domain exhibited other inflammatory conditions such as abscesses (n=7), cellulitis (n=4), arthritis (n=3), cholecystitis (n=1), and ulcers (n=1). Inflammatory conditions in XLA may be associated with dysregulation of certain toll-like receptor (TLR) pathways (41), a disturbed transcription of TLR negative regulators (42) or an increased activation of NLRP3 inflammasome in monocytes (43). Respiratory infections such as pneumonia was the main clinical presentation in this cohort, which was in line with previous studies (5, 21, 35, 44). Among those patients with pneumonia, 10 patients developed bronchiectasis as diagnosed by high-resolution computed tomography (HRCT). Regular monitoring of respiratory function by pulmonary function tests and/or HRCT should be implemented in XLA patients with pneumonia to prevent progression to permanent lung damage (45). Patients with XLA are often susceptible to pyogenic infections caused by pyogenic bacteria such as *Pseudomonas aeruginosa, Staphylococcus aureus, Streptococcus pneumoniae*, and *Haemophilus influenzae* (4, 5, 23, 46). In our cohort, the pyogenic infections were also caused by these pathogens. On the contrary, mycobacterial infection that is generally defended by cell-mediated immunity, is rarely seen in XLA patients. However, it could still be observed in a minority of XLA patients from countries which are endemic for tuberculosis such as Argentina (6), India (5) and our country. Emerging evidence suggested that humoral-mediated immunity contributed significantly to the development of immune responses to the tubercle bacillus, hence reducing the mycobacterial burden (47). On the other hand, severe acute respiratory syndrome coronavirus 2 (SARS-CoV-2) causes coronavirus disease 2019 (COVID-19) in millions globally, patients may exhibit mild-to-severe symptoms. In this cohort, four patients suffered from COVID-19 infection with either mild or no symptoms. Previous study reported XLA patients may present with a mild clinical course of COVID-19 (48). It was shown that XLA patients did not produce antibody, but developed spike SARS-CoV-2 T-cells following BNT162b2 vaccine (49). Therefore, XLA patients are likely protected by cell-mediated immunity.

It is described that Bruton’s tyrosine kinase (BTK) is not only indispensable to the biology and function of B cells but also other innate myeloid immune cells such as monocytes, macrophages (50), neutrophils (51), and dendritic cells (52). In particular, BTK plays a role in regulating FcγR-mediated cytokine production (53), chemotaxis (54), as well as NLRP3 inflammasome activation in the monocytes (43). In the present study, all patients except P10 had a low percentage (<2%) of B cells. Considering BTK protein is expressed in most B cells and myeloid cells such as monocytes (16), flow cytometric analysis of cytoplasmic BTK protein in monocytes is useful for evaluation of BTK deficiency in XLA patients that lack peripheral B cells. In this cohort, thirteen patients had absent or minimal intracellular BTK expression (0-15%) in the monocytes. A complete BTK protein deficiency was observed in two patients (P6 and P7) with missense mutations affecting the kinase domain of BTK protein. On the other hand, eight patients had detectable levels of BTK expression (18-64% positive) in the monocytes. Of these patients, two patients had missense mutations (P13 and P18), and six patients had splicing mutations (P2, P16, P17, P19, P20 and P21). It was described that a mutation affecting the invariant splice site often leads to alternative splicing, resulting in exon skipping, intron inclusion, use of a cryptic splice site, leaky splicing or pseudo-exon inclusion (55, 56). In this report, we observed a splicing mutation (c.1103-2A>G) in genomic DNA of a pair of twins (P19 and P20) yielded several mRNA transcripts. Further sequencing analysis revealed that the point mutation disrupted the use of authentic acceptor splice site in the intron 12, and activated the use of a cryptic splice site either in the intron 12 or exon 13, resulting in the inclusion of a 170 basepair intron or a 13 basepair exon deletion into its *BTK* mRNA transcript, respectively.

A flow cytometric assay is also informative for detecting cellular mosaicism of BTK expression in the female carriers. In this cohort, flow cytometric analysis clearly showed the mosaic pattern of BTK expression in monocytes of some female carriers, demonstrating they have both BTK-positive and BTK-negative monocytes. However, some female carriers exhibited indistinct mosaic BTK expression whereby the dimly expressed BTK monocytes unified with those non-BTK monocytes. Furthermore, flow cytometric assay demonstrated normal BTK expression (77-97% positive) in monocytes of four mothers (family 3, 9, 11 and 12). In such cases, a *BTK* genetic carrier testing should be performed to confirm the carrier status. The mosaicism in the female carrier’s monocytes can be explained with X-inactivation. X-inactivation is important for sex chromosome dosage compensation in females to ensure a balanced expression of X-linked genes between females and males. The choice of which of the two X’s inactivated is random, but not all females have equal ratio of cells with either the paternal or maternal X chromosome inactivated (57). Random X-inactivation may result in varied pattern of mosaicism (58), as exhibited by the BTK protein expression of female carriers in this study. On the other hand, *BTK* genetic carrier testing revealed that the mother (family 12) carried both normal and mutated alleles despite having normal BTK protein expression, suggesting skewed X-inactivation of the mutated X-chromosome in monocytes.

The combination of genetic analysis using cDNA and genomic DNA may provide insights into *BTK* RNA splicing mechanism. For instance, a large deletion spanning exon 8-10 was detected from the cDNA analysis of two brothers (P3 and P4). The exon skipping was speculated to be resulted from a splicing mutation involving intron 7 or intron 10. But no amplicon was obtained when amplifying their genomic DNAs using specific primer sets that flank the splice sites of exon 8, 9, and 10 respectively. A further investigation involving amplification of the intron 6-12 of the *BTK* gene revealed that both patients’ PCR products were larger in size (~5.6kb) as compared to the control (~4.5kb). The subsequent cloning and sequencing analysis revealed a large indel mutation involving the loss of invariant splice sites in the intron 7-10, which disrupted the splicing mechanism. As observed from the cDNA analysis, this mutation resulted in the deletion of exon 8-10 which code for the SH2 and SH3 domain of BTK protein. It is known that the BTK function was regulated by an important autophosphorylation site, i.e. Tyr223 residue in the SH3 domain (59). This mutation was predicted to be deleterious and evidenced by a minimal BTK expression (1% positive) on both patients’ monocytes. Although both siblings carried the similar mutation, they exhibited distinctly different clinical phenotypes. The elder brother had neurologic deterioration of unknown cause and later died of sepsis at the age of 16-year-old following severe pneumonia, while the younger brother had only mild symptom such as recurrent lymphadenitis. He has been well without bronchiectasis or frequent infections after receiving regular immunoglobulin infusion since he was five years old.

Up to year 2023, more than 2,300 variants were documented in Global Variome shared Leiden Open Variation Database (LOVD3) (https://databases.lovd.nl/shared/genes/BTK). These variants scattered over the *BTK* gene. The genetic profiles of 22 XLA patients in this current cohort also showed that the identified mutations spanning all domains in BTK, except TH domain. Similar with previously reported cohorts (21, 44, 60), most of the mutations were found in the kinase domain, emphasizing the significant function of the kinase domain in the B cell development. Of the mutations affecting the kinase domain, the residues Arg520 and Arg525 are arginine-coding CpG dinucleotides that were reported as the frequent mutation sites (61–64). One of our patients carried p.Arg520Leu mutation and two patients carried p.Arg525* mutation. The p.Arg520Leu mutation would disrupt the salt-bridge interaction with Glu445 and Asp579, while the p.Arg525* mutation would disrupt the salt-bridge interaction with Asp521 and Glu589, thereby destabilizing the activation loop. Besides that, the mutation sites (Arg520 and Arg525) are also located very close to the active site, Tyr551 in the kinase domain of BTK protein (65), therefore, mutation in these sites would affect the function of the BTK protein.

In this work, more than two thirds of the pathogenic variants are caused by splicing defects, frameshift mutations or premature stop codons. The most frequently recurrent mutation sites in *BTK* are the invariant splice site in the intron 9 (c.839 + 1G>C). This mutation was observed in three patients from two unrelated families, resulting in skipping of the exon 9. Mutation at this splice site has been reported in several studies (66, 67). On the other hand, the c.215del is the only one mutation affecting the PH domain in this study. Repetitive DNA sequence, for example, a stretch of seven adenosine nucleotide at positions 209-215 of *BTK* gene, is vulnerable to replication slippage during DNA replication (68). During the misalignment, either deletion or addition of nucleotide happens (35, 62, 69) resulting in a non-functional protein. This mutation was also found to be *de novo* in our patient. Other than this mutation, two other point mutations (c.1181C>A and c.1559G>T) were also found to be *de novo*. Therefore, it is noteworthy to mention that the *BTK* mutation could occur sporadically (3, 21, 62, 63, 70, 71), although most of the patients inherited the mutation from their mothers.

## Conclusion

To the best of our knowledge, this is the first XLA cohort describing clinical, immunological and genetic profiles from Malaysia. Twenty-two patients with B-cell deficiency and hypogammaglobulinemia were diagnosed with XLA using *BTK* genetic analysis. Variable clinical phenotypes including atypical phenotypes were observed in some patients. Flow cytometric analysis of monocyte BTK protein expression is a rapid and useful tool in detecting XLA patients and the carriers, but detectable BTK protein expression can be observed in some patients with substitution or splice site mutations. Therefore, *BTK* mutational analysis is needed in providing the definitive diagnosis of XLA.

## Data availability statement

The raw data supporting the conclusions of this article will be made available by the authors, without undue reservation.

## Ethics statement

The studies involving humans were approved by Medical Research and Ethics Committee, Ministry of Health Malaysia. The studies were conducted in accordance with the local legislation and institutional requirements. Written informed consent for participation in this study was provided by the participants’ legal guardians/next of kin. Written informed consent was obtained from the individual(s), and minor(s)’ legal guardian/next of kin, for the publication of any potentially identifiable images or data included in this article.

## Author contributions

CTC designed the study, performed the research, analyzed data and wrote the manuscript. IHI, KCC, LMN, AK, AHAL, SSG, NHR, KKT, CS, CMC, and SASM recruited and managed the patient, and provided clinical information. MFB, ASZ and SBM analyzed data, reviewed andrevised the manuscript. SNHSY performed experiment, reviewed and revised the manuscript. AMR designed, supervised the study, reviewed and revised the manuscript. All authors contributed to the article and approved the submitted version.
