# Supplementary material for: Clinical features and mutational analysis of X-linked agammaglobulinemia patients in Malaysia
Source: Front Immunol. 2023 Sep 22;14:1252765. doi: 10.3389/fimmu.2023.1252765 (PMC10560089; doi:10.3389/fimmu.2023.1252765)
Supplement: Supplementary file 1 [file Table_1.docx]

Supplementary Table 1. Monocyte BTK expression evaluation and genetic screening of the patient’s family members

| **Family no.** | **Subject ID (B/M/S)** | **Monocyte BTK expression (%)** | **Monocyte BTK expression pattern** | **Genotype** |
| --- | --- | --- | --- | --- |
| F1 | M | 69 | Mosaic | HT |
|  | S | 18 | Mosaic | HT |
| F2 | M | 13 | Mosaic | NE |
|  | S | 85 | Normal | WT |
| F3 | M | 94 | Normal | WT |
|  | S | 98 | Normal | WT |
|  | S | 94 | Normal | WT |
| F4 | M | 51 | Mosaic | HT |
| F5 | M | 32 | Mosaic | HT |
|  | S | 43 | Mosaic | HT |
|  | M | 28 | Mosaic | HT |
| F8 | M | 71 | Mosaic | HT |
|  | B | 98 | Normal | WT |
| F9 | M | 77 | Normal | WT |
| F10 | M | 76 | Mosaic | HT |
| F11 | M | 97 | Normal | WT |
|  | B | 99 | Normal | NE |
| F12 | M | 85 | Normal | HT |
|  | S | 91 | Normal | WT |
|  | B | 98 | Normal | WT |
|  | B | 98 | Normal | WT |
|  | S | 95 | Normal | WT |
| F13 | M | 54 | Mosaic | HT |
|  | S | 55 | Mosaic | HT |
|  | S | 41 | Mosaic | HT |
|  | S | 41 | Mosaic | HT |
|  | S | 56 | Mosaic | HT |
|  | S | 33 | Mosaic | HT |
| F14 |  | 81 | Mosaic | HT |
| F15 | M | 51 | Mosaic | HT |
|  | S | 99 | Normal | NE |
| F16 | M | 47 | Mosaic | HT |

B, brother; F, family; M, mother; HT, heterozygous; NE, not examined; P, patient; S, sister; WT, wild type.

Supplementary Table 2. COVID-19 infection in XLA

| Patient ID | COVID-19 |
| --- | --- |
| P1 | No |
| P2 | No |
| P3 | Deceased before COVID-19 pandemic |
| P4 | No |
| P5 | COVID-19 category 2 |
| P6 | NA |
| P7 | NA |
| P8 | No |
| P9 | NA |
| P10 | Deceased before COVID-19 pandemic |
| P11 | No |
| P12 | No |
| P13 | NA |
| P14 | No |
| P15 | COVID-19 category 1 |
| P16 | No |
| P17 | COVID-19 category 2 |
| P18 | No data |
| P19 | NA |
| P20 | COVID-19 category 2 |
| P21 | NA |
| P22 | No data |

NA, Not available.

Supplementary Table 3. *BTK* mutation analysis of XLA patients

| **Family no.** | **Patient ID** | **Localization** | **Domain affected** | **Nucleotide change in genomic DNA** | **Nucleotide change in cDNA level** | **Predicted codon change** | **Type of mutation** | **Mother status^#^** | **Novel/References** |
| --- | --- | --- | --- | --- | --- | --- | --- | --- | --- |
| F1 | P1 | Intron 9 | SH3 | c.839+1G>C | c.777_839del | p.Gln260_Glu280del | Splicing | Carrier | (1,2) |
| F1 | P2 | Intron 9 | SH3 | c.839+1G>C | c.777_839del | p.Gln260_Glu280del | Splicing | Carrier |  |
| F2 | P3 | Intron 7-10 | SH3,SH2 | NC_000023.11:g.101358809_101361213delins[(302);NG_052969.1:g.788322_791554;GT] | c.589_894del | p.Ile197_Glu298del | Indel | NE | Novel |
| F2 | P4 | Intron 7-10 | SH3,SH2 | NC_000023.11:g.101358809_101361213delins[(302);NG_052969.1:g.788322_791554;GT] | c.589_894del | p.Ile197_Glu298del | Indel | NE | Novel |
| F3 | P5 | Exon 14 | Kinase | g.34273C>A | c.1181C>A | p.Ser394* | Nonsense mutation | NMD | Novel |
| F4 | P6 | Exon 13 | Kinase | g.33712A>G | c.1173_1177del | p.Tyr392Ilefs*5 | Nonsense mutation | Carrier | (3) |
| F5 | P7 | Exon 14 & Intron 14 | Kinase | g.34430_34447del | c.1338_1349delins[NG_009616.1:  g.34448_34531] | p.Ala446_Asn451ins28 | Deletion | Carrier | (2) |
| F5 | P8 | Exon 14 & Intron 14 | Kinase | g.34430_34447del | c.1338_1349delins[NG_009616.1:  g.34448_34531] | p.Ala446_Asn451ins28 | Deletion | Carrier |  |
| F6 | P9 | Exon 18 | Kinase | c.1888A>T | c.1888A>T | p.Met630Leu | Missense mutation | NE | (2,4) |
| F7 | P10 | Intron 14 | Kinase | c.1349+5G>A | c.1349_1350ins[GTGAA;  NG_009616.1:g.34447_34531] | p.Asn451* | Splicing | NE | (5) |
| F8 | P11 | Exon 16 | Kinase | g.36537C>T | c.1573C>T | p.Arg525* | Nonsense mutation | Carrier | Reported according to LOVD3 database |
| F8 | P12 | Exon 16 | Kinase | g.36537C>T | c.1573C>T | p.Arg525* | Nonsense mutation | Carrier | Reported according to LOVD3 database |
| F9 | P13 | Exon 15 | Kinase | g.35166G>T | c.1559G>T | p.Arg520Leu | Missense mutation | NMD | Novel |
| F10 | P14 | Exon 19 | Kinase | g.41282G>A | c.1922G>A | p.Arg641His | Missense mutation | Carrier | (6) |
| F11 | P15 | Exon3 | PH | g.16664del | c.215del | p.Asn72Ilefs*49 | Deletion | NMD | (7–9) |
| F12 | P16 | Intron 12 & Exon 13 | SH2 | g.33640-33661delinsTG | c.1103_1129del | p.Gly368_Pro376del | Splicing | Carrier | Novel |
| F12 | P17 | Intron 12 & Exon 13 | SH2 | g.33640-33661delinsTG | c.1103_1129del | p.Gly368_Pro376del | Splicing | Carrier | Novel |
| F13 | P18 | Exon 18 | Kinase | g.37969C>G | c.1846C>G | p.Leu616Val | Missense mutation | Carrier | (10) |
| F14 | P19 | Intron 12 | SH2 | c.1103-2A>G | c.1103_1115del ; c.1102_1103ins[NG_009616.1:  g.33472_33639;GG] | p.Leu369Serfs*30; p.Gly368Valfs*32 | Splicing | Carrier | Novel |
| F14 | P20 | Intron 12 | SH2 | c.1103-2A>G | c.1103_1115del ; c.1102_1103ins[NG_009616.1:  g.33472_33639;GG] | p.Leu369Serfs*30; p.Gly368Valfs*32 | Splicing | Carrier | Novel |
| F15 | P21 | Intron 9 | SH3 | c.839+1G>C | c.777_839del | p.Gln260_Glu280del | Splicing | Carrier | (1) |
| F16 | P22 | Exon 16 | Kinase | g.36544_36545del | c.1580_1581del | p.Cys527Phefs*9 | Deletion | Carrier | (11) |

^#^The carrier status of the mother was confirmed by *BTK* genetic carrier screening; NMD, no mutation detected; NE, not examined.

References:

1. Chear CT, Gill HK, Dhaliwal JS, Bujang N, Ripen AM, Mohamad SB. A novel Bruton’s tyrosine kinase gene (BTK) invariant splice site mutation in a Malaysian family with X-linked agammaglobulinemia. *Asian Pac J Allergy Immunol* (2013) 31: doi: 10.12932/AP0304.31.4.2013

2. Mirsafian H, Ripen AM, Leong W-M, Chear CT, Mohamad SB, Merican AF. Transcriptome profiling of monocytes from XLA patients revealed the innate immune function dysregulation due to the BTK gene expression deficiency. *Sci Rep* (2017) 7:6836. doi: 10.1038/s41598-017-06342-5

3. Chear CT, Ripen AM, Mohamed SAS, Dhaliwal JS. A novel BTK gene mutation creates a de-novo splice site in an X-linked agammaglobulinemia patient. *Gene* (2015) 560:245–248. doi: 10.1016/j.gene.2015.02.019

4. Ali A, Wahab AA, Latiff AHA, Ismail IH, Zaki FM, Borhanuddin BK, Noh LM. Clinical and laboratory observation on immunoglobulin replacement therapy switching from an intravenous to a subcutaneous route in a Malaysian X-linked agammaglobulinemia patient. *Med J Malaysia* (2022) 77:95–97.

5. Fiorini M, Franceschini R, Soresina A, Schumacher R-F, Ugazio AG, Rossi P, Plebani A, Notarangelo LD, Italian XLA Collaborative Group of the Italian Pediatric Hematology-Oncology Association (AIEOP). BTK: 22 novel and 25 recurrent mutations in European patients with X-linked agammaglobulinemia. *Hum Mutat* (2004) 23:286–286. doi: 10.1002/humu.9219

6. Speletas M, Kanariou M, Kanakoudi-Tsakalidou F, Papadopoulou-Alataki E, Arvanitidis K, Pardali E, Constantopoulos A, Kartalis G, Vihinen M, Sideras P. Analysis of Btk Mutations in Patients with X-Linked Agammaglobulinaemia (XLA) and Determination of Carrier Status in Normal Female Relatives: a Nationwide Study of Btk Deficiency in Greece. *Scand J Immunol* (2001) 54:321–327.

7. Aghamohammadi A, Fiorini M, Moin M, Parvaneh N, Teimourian S, Yeganeh M, Goffi F, Kanegane H, Amirzargar AA, Pourpak Z, et al. Clinical, Immunological and Molecular Characteristics of 37 Iranian Patients with X-Linked Agammaglobulinemia. *Int Arch Allergy Immunol* (2006) 141:408–414. doi: 10.1159/000095469

8. Danielian S, El-Hakeh J, Basilico G, Oleastro M, Rosenzweig S, Feldman G, Berozdnik L, Galicchio M, Gallardo A, Giraudi V, et al. Bruton tyrosine kinase gene mutations in Argentina. *Hum Mutat* (2003) 21:451–451. doi: 10.1002/humu.9131

9. Abolhassani H, Vitali M, Lougaris V, Giliani S, Parvaneh N, Parvaneh L, Mirminachi B, Cheraghi T, Khazaei H, Mahdaviani SA, et al. Cohort of Iranian Patients with Congenital Agammaglobulinemia: Mutation Analysis and Novel Gene Defects. *Expert Rev Clin Immunol* (2016) 12:479–486. doi: 10.1586/1744666X.2016.1139451

10. Tóth B, Volokha A, Mihas A, Pac M, Bernatowska E, Kondratenko I, Polyakov A, Erdos M, Pasic S, Bataneant M. Genetic and demographic features of X-linked agammaglobulinemia in eastern and central Europe: a cohort study. *Mol Immunol* (2009) 46:2140–2146.

11. Chear CT, Nallusamy R, Chan KC, Mohd Tap R, Baharin MF, Syed Yahya SNH, Krishnan PB, Mohamad SB, Ripen AM. Atypical Presentation of Severe Fungal Necrotizing Fasciitis in a Patient with X-Linked Agammaglobulinemia. *J Clin Immunol* (2021) doi: 10.1007/s10875-021-01017-3

Supplementary Table 4. *In silico* functional impact predictions of the identified mutations

| **Patient ID** | **Mutation** | **SIFT** | **Polyphen-2** | **MutationTaster2** | **Provean** | **Varsome** |
| --- | --- | --- | --- | --- | --- | --- |
| P1 | c.777_839del^£^; Q260_E280del^¥^ | - | - | - | Deleterious | Pathogenic |
| P2 | c.777_839del^£^; Q260_E280del^¥^ | - | - | - | Deleterious | Pathogenic |
| P3 | c.589_894del^£^; I197_E298del^¥^ | - | - | - | Deleterious | Pathogenic |
| P4 | c.589_894del^£^; I197_E298del^¥^ | - | - | - | Deleterious | Pathogenic |
| P5 | c.1181C>A^ɤ,£^ | - | - | Disease causing | - | Pathogenic |
| P6 | c.11173_1177del^£^ | - | - | - | - | Pathogenic |
| P7 | c.1338_1349delinsTATAGCCCAAACTCAACTCTCAATCTATTTGCTGGAGTCTAGGAATTCACACAACAACCCACTGAGGCTTAAAGATGACTTACA^£^; A446_N451delinsIAQTQLSIYLLESRNSHNNPLRLKDDLQ^¥^ | - | - | - | Deleterious | Likely pathogenic |
| P8 | c.1338_1349delinsTATAGCCCAAACTCAACTCTCAATCTATTTGCTGGAGTCTAGGAATTCACACAACAACCCACTGAGGCTTAAAGATGACTTACA^£^; A446_N451delinsIAQTQLSIYLLESRNSHNNPLRLKDDLQ^¥^ | - | - | - | Deleterious | Likely pathogenic |
| P9 | c.1888A>T^ɤ,£^; M630L^§,ρ,¥^ | Affect protein function | Possibly damaging | Disease causing | Deleterious | Pathogenic |
| P10 | c.1349+5G>A^£^ | - | - | - | - | Pathogenic |
| P11 | c.1573C>T^ɤ,£^ | - | - | Disease causing | - | Pathogenic |
| P12 | c.1573C>T^ɤ,£^ | - | - | Disease causing | - | Pathogenic |
| P13 | c.1559G>T^ɤ,£^; R520L^§,ρ,¥^ | Affect protein function | Probably damaging | Disease causing | Deleterious | Pathogenic |
| P14 | c.1922G>A^ɤ,£^; R641H^§,ρ,¥^ | Affect protein function | Probably damaging | Disease causing | Deleterious | Pathogenic |
| P15 | c.215del^ɤ,£^ | - | - | Disease causing |  | Pathogenic |
| P16 | c.1103_1129del^ɤ,£^; G368_P376del^¥^ | - | - | Disease causing | Deleterious | Pathogenic |
| P17 | c.1103_1129del^ɤ,£^; G368_P376del^¥^ | - | - | Disease causing | Deleterious | Pathogenic |
| P18 | c.1846C>G^ɤ,£^; L616V^§,ρ,¥^ | Affect protein function | Probably damaging | Disease causing | Deleterious | Likely pathogenic |
| P19 | c.1103_1115del^ɤ,£^ | - | - | Disease causing | - | Pathogenic |
| P20 | c.1103_1115del^ɤ,£^ | - | - | Disease causing | - | Pathogenic |
| P21 | c.777_839del^£^; Q260_E280del^¥^ | - | - | - | Deleterious | Pathogenic |
| P22 | c.1580_1581del^ɤ,£^ | - | - | Disease causing | - | Pathogenic |

The mutation nomenclature used as the input for SIFT^§^, Polyphen-2^ρ^, MutationTaster2^ɤ^, Provean^¥^ and Varsome^£^ prediction tool; -, not applicable.
